# Quantifying Radiosensitization of PSMA-Targeted Gold Nanoparticles on Prostate Cancer Cells at Megavoltage Radiation Energies by Monte Carlo Simulation and Local Effect Model

**DOI:** 10.3390/pharmaceutics14102205

**Published:** 2022-10-17

**Authors:** Ryder M. Schmidt, Daiki Hara, Jorge D. Vega, Marwan B. Abuhaija, Wensi Tao, Nesrin Dogan, Alan Pollack, John C. Ford, Junwei Shi

**Affiliations:** 1 Department of Radiation Oncology, Miller School of Medicine, University of Miami, Miami, FL 33136, USA; 2 Department of Biomedical Engineering, University of Miami, Coral Cables, FL 33146, USA

**Keywords:** gold nanoparticle, nanoparticle enhanced radiation therapy, Monte Carlo, local effect model, dose enhancement ratio, sensitization enhancement ratio

## Abstract

Active targeting gold nanoparticles (AuNPs) are a very promising avenue for cancer treatment with many publications on AuNP mediated radiosensitization at kilovoltage (kV) photon energies. However, uncertainty on the effectiveness of AuNPs under clinically relevant megavoltage (MV) radiation energies hinders the clinical translation of AuNP-assisted radiation therapy (RT) paradigm. The aim of this study was to investigate radiosensitization mediated by PSMA-targeted AuNPs irradiated by a 6 MV radiation beam at different depths to explore feasibility of AuNP-assisted prostate cancer RT under clinically relevant conditions. PSMA-targeted AuNPs (PSMA-AuNPs) were synthesized by conjugating PSMA antibodies onto PEGylated AuNPs through EDC/NHS chemistry. Confocal fluorescence microscopy was used to verify the active targeting of the developed PSMA-AuNPs. Transmission electron microscopy (TEM) was used to demonstrate the intracellular biodistribution of PSMA-AuNPs. LNCaP prostate cancer cells treated with PSMA-AuNPs were irradiated on a Varian 6 MV LINAC under varying depths (2.5 cm, 10 cm, 20 cm, 30 cm) of solid water. Clonogenic assays were carried out to determine the in vitro cell survival fractions. A Monte Carlo (MC) model developed on TOPAS platform was then employed to determine the nano-scale radial dose distribution around AuNPs, which was subsequently used to predict the radiation dose response of LNCaP cells treated with AuNPs. Two different cell models, with AuNPs located within the whole cell or only in the cytoplasm, were used to assess how the intracellular PSMA-AuNP biodistribution impacts the prostate cancer radiosensitization. Then, MC-based microdosimetry was combined with the local effect model (LEM) to calculate cell survival fraction, which was benchmarked against the in vitro clonogenic assays at different depths. In vitro clonogenic assay of LNCaP cells demonstrated the depth dependence of AuNP radiosensitization under clinical megavoltage beams, with sensitization enhancement ratio (SER) of 1.14 ± 0.03 and 1.55 ± 0.05 at 2.5 cm depth and 30 cm depth, respectively. The MC microdosimetry model showed the elevated percent of low-energy photons in the MV beams at greater depth, consequently resulting in increased dose enhancement ratio (DER) of AuNPs with depth. The AuNP-induced DER reached ~5.7 and ~8.1 at depths of 2.5 cm and 30 cm, respectively. Microdosimetry based LEM accurately predicted the cell survival under 6 MV beams at different depths, for the cell model with AuNPs placed only in the cell cytoplasm. TEM results demonstrated the distribution of PSMA-AuNPs in the cytoplasm, confirming the accuracy of MC microdosimetry based LEM with modelled AuNPs distributed within the cytoplasm. We conclude that AuNP radiosensitization can be achieved under megavoltage clinical radiotherapy energies with a dependence on tumor depth. Furthermore, the combination of Monte Carlo microdosimetry and LEM will be a valuable tool to assist with developing AuNP-aided radiotherapy paradigm and drive clinical translation.

## 1. Introduction

Cancer continues to be a major public health problem around the world and in the United States. Prostate cancer is one of the most common types of cancer with an estimated 268,490 new cases and 34,500 deaths in the United States for 2022 alone [1]. Approximately 50% of all cancer patients and 34% of prostate cancer patients will undergo radiation therapy (RT) during the course of treatment [2,3]. Radiation therapy aims to irradiate the target (tumor) while minimizing the dose to surrounding healthy tissue. Engineering advancements to external beam radiation therapy (EBRT) such as intensity modulated radiation therapy (IMRT) and volumetric modulated arc therapy (VMAT) have improved the conformity of treatments, increasing the tumor control probability (TCP) and decreasing the normal tissue complication probability (NTCP). However, further alleviating the side effects induced by the dose to surrounding organs at risk (OAR) remains a challenge. Side effects of EBRT for prostate cancer include frequent urination, diarrhea, rectal bleeding, abdominal cramps, fatigue and more. Radiosensitizers have been proposed as a means to increase EBRT efficacy, improving treatment outcomes while mitigating unwanted side effects.

In recent years, the use of nanoparticles (NPs) as radiosensitizers has gained a great deal of interest in the field of cancer radiotherapy [4,5]. High atomic number (high-Z) metallic NPs have shown an ability to locally enhance radiation doses, increasing the DNA damage in close proximity to the NPs due to the increased photoelectric interaction and increased Auger electron production [6,7]. As a type of metal NP, gold nanoparticles (AuNPs) attract wide attention due to their special merits including, easily modified surface characteristics, relative chemical and biological stability, and ease of fabrication [4,8,9]. Other metal NPs have also been used as radiosensitizers such as Gadolinium NPs (GdNPs) and Platinum NPs (PtNPs) [10,11]. However, as a versatile nano-platform, AuNPs also demonstrates its prominent theranostic performance in molecular imaging, photothermal therapy, and drug delivery [12]. AuNPs will naturally accumulate within the tumor due to leaky vasculature and poor lymphatic clearance within the tumor, known as enhanced permeability and retention (EPR) [13,14]. Moreover, further enhanced AuNP accumulation within the tumor can be achieved when using actively targeting AuNPs, as was validated with PSMA-targeted AuNPs (PSMA-AuNPs) [15]. Actively targeted AuNPs results in increased therapeutic ratio by achieving greater concentrations of nanoparticles within the tumor than in the surrounding healthy tissue [16], improving the efficacy of AuNP-assisted RT.

The first study using a AuNP as a radiosensitizer was conducted by Hainfeld et al. in 2004, where mice bearing subcutaneous EMT-6 mammary carcinomas treated with radiation and AuNPs had a higher survival rate than among mice treated with radiation alone [17]. This successful study spurred a wave of research regarding AuNPs and their ability to improve radiation treatment outcomes. AuNP mediated radiosensitization under photon radiation has been widely studied in vivo, in vitro and in silico [4,5,9,18,19,20,21,22,23]. The degree of radiosensitization observed depends on many factors such as AuNP size, biodistribution, cancer type, and energies of the irradiation source. Kilovoltage (kV) beams are expected to have the highest degree of sensitization due the high probability of a photoelectric interaction (*P_PE_*) between kV photons and Gold, as shown in Equation (1):

(1) $$P_{PE}∼{\left(\frac{Z}{E}\right)}^{3}$$

where *E* is the photon energy and *Z* is the atomic number (*Z* = 79 for Gold). The resultant photoelectrons and/or Auger electrons deposit dose locally, giving rise to a localized dose enhancement inducing more cell killing. For megavoltage (MV) photons the dominant interactions with gold and water are Compton scattering and pair production which have a relatively low dependence on *Z*. MV beams have a low photoelectric interaction probability with little difference between gold and tissue; therefore, MV beams are not expected to result in a localized dose enhancement. However, clinical irradiators (e.g., LINACs) generate MV beams that are polyenergetic and contain many low energy (kV) photons in addition to the high energy (MV) photons. Typically, LINACs contain flattening filters (FF) to achieve the desired dose profile. However, the use of FFs also attenuates many of the low energy photons. In the absence of a flattening filter, i.e., flattening filter free (FFF), a greater proportion of the beam is at kV energies, indicating a possible enhancement in photoelectric interactions with AuNPs. Nevertheless, a high initial kV component of the beam will be attenuated/absorbed while passing through tissue, resulting in a limited effect on the extent of AuNP-induced radiosensitization for deep seated tumors. Additionally, as MV beams traverse matter, scatter and attenuation also contribute to an increased proportion of low energy photons, again indicating a possible increase in the photoelectric interactions.

Several in vitro and in vivo studies have validated AuNP-mediated radiosensitization under 6 MV radiation beams. For example, Wolfe et al. found that PC3 prostate cancer cells treated with Au nanorods (AuNRs) and 6 MV radiation in vitro had significant radiosensitization [24]. An in vitro study by Yang et al. found that MDA-MB-231 cells treated with AuNPs and 6 MV radiation resulted in a 19% decrease in cell survival when compared to cells treated with radiation alone [25]. Wolfe et al. also found that PC3 prostate cancer cells treated with AuNRs and radiation in vivo had significant radiosensitization [24]. While the majority of in vitro and in vivo studies report promising results, the quantification of dose enhancement based on Monte Carlo (MC) demonstrate disparities. For example, McMahon et al. evaluated the nanodosimetric effects of AuNPs under 6 MV radiation via MC simulations and found significant sensitization of MDA-MB-231 breast cancer cells with sensitization enhancement ratio (SER) of 1.24 ± 0.05 at 5 cm depth [1]. On the other hand, a macroscopic study conducted by Gray et al. showed that AuNPs under 6 MV radiation exhibited no dose enhancement [26]. These disparities highlight the complexities of MC simulations in nanoparticle enhanced radiation therapy. A major factor influencing the AuNP-induced dose enhancement in MC simulations is whether the dosimetry is calculated on a macroscopic or micro-/nano-scopic scale. Most macroscopic simulations use condensed history (CH) physics lists whereas nanoscopic simulations often use track structure (TS) physics lists. CH physics lists group many steps into a single step, which creates a fast simulation with the tradeoff of lower spatial resolution; TS physics lists follow each particle step-by-step, creating very accurate and high-resolution results with the tradeoff of slow speeds. It has been shown that on a macroscopic scale, the dose enhancement from AuNPs appears to be negligible. However, when nanoscopic simulations are employed, a clear dose spike is present near the AuNP [2]. Geant4 (GEometry ANd Tracking) is an open source MC simulation toolkit with both CH and TS physics models [27]. Geant4 and its DNA-scale radiobiological extension, Geant4-DNA [28], are wrapped in a user-friendly parameter file-based system, TOPAS (TOol for PArticle Simulation) and TOPAS-nBio, respectively [29,30]. TOPAS and TOPAS-nBio have been successfully used by many research groups for AuNP mediated dose enhancement studies [8,31,32,33]. The MC simulation of AuNP microdosimetry in this study was performed on the TOPAS platform.

While MC simulations can provide high resolution dose distributions in the presence of AuNPs, translating this information to the context of in vitro and in vivo experiments is still crucial to guide the design of AuNP-assisted radiotherapy. Using MC-simulated dose distributions in conjunction with the local effect model (LEM), the biological endpoint of cell survival fraction can be calculated. The LEM was originally developed for heavy charged particle therapy such as carbon ion therapy where the dose distribution is spatially inhomogeneous due to the high linear energy transfer (LET) particle tracks [34]. This model was also found to work for the spatially inhomogeneous dose distribution caused by AuNPs and has been successfully used by several groups to model cell survival curves under AuNP mediated irradiation [20,35].

In this study, a MC model developed on TOPAS was used to determine the nano-scale radial dose distribution around an AuNP, which was subsequently used to predict the radiation dose response of LNCaP cells accumulated with AuNPs. Two different cell models, with AuNPs located within the whole cell or only in cytoplasm, were used to assess how the intracellular PSMA-AuNP biodistribution impacts the prostate cancer radiosensitization. Then, MC-based microdosimetry was combined with the local effect model (MC-LEM) to calculate cell surviving fraction, which was benchmarked against the in vitro clonogenic assays at different depths. To the best of our knowledge, this is the first study to systematically investigate the impact of depth on AuNP-induced radiosensitization of prostate cancer under clinical megavoltage radiation energies in vitro. Furthermore, by modeling the cell geometry with a realistic AuNP distribution, the MC-LEM model demonstrated its accuracy in predicting the cancer cell kill.

## 2. Materials and Methods

### 2.1. In Vitro Experiment

Cell experiments were carried out with LNCaP prostate cancer cells (ATCC, Rockville, MD, USA) maintained in RPMI1640 (Thermo Fisher Scientific, Waltham, MA, USA) containing 10% fetal bovine serum and 1% penicillin-streptomycin. Cell cultures were maintained at 37 °C and 5% (*v*/*v*) CO_2_. Commercially available 15 nm PEGylated AuNPs (Creative Diagnostics, New York, NY, USA) were used in this study. To realize active targeting for LNCaP cells, a PSMA expressing prostate cancer cell line, anti-PSMA antibodies (Creative Diagnostics) were coupled to PEGylated AuNPs through 1-ethyl-3-(-3-dimethylaminopropyl) carbodiimide hydrochloride (EDC) and N-hydroxysuccinimide (NHS) EDC/NHS chemistry. Confocal fluorescence microscopic imaging was performed to verify the active targeting of the developed PSMA-targeted AuNPs, where AuNPs were tagged with AlexaFluor 488 TFP ester (Thermo Fisher Scientific, Waltham, MA, USA) prior to cell treatment. Transmission electron microscopy (TEM) was performed to identify the intracellular biodistribution of AuNPs in prostate cancer cells. The details on the synthesis of PSMA-targeted AuNPs, fluorescence microscopy and TEM imaging can be found in our previous publication [36].

**Figure 1:**
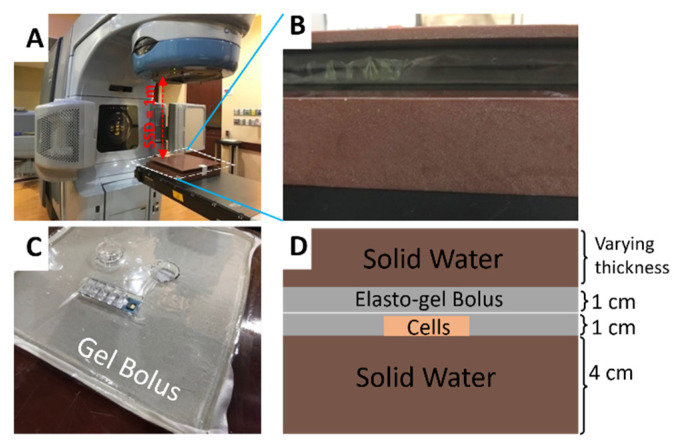
(**A**) Experimental in vitro cell irradiation on LINAC. (**B**) Bolus between solid water backscatter and solid water build-up from side view. (**C**) Circular cell cutouts within elasto-gel bolus. (**D**) Diagram showing cells, bolus and solid water used for in vitro cell irradiations.

The in vitro experimental setup is shown in Figure 1. 24 h prior to irradiation, LNCaP cancer cells were treated with 250 µg/mL PSMA-targeted AuNPs in serum-free RPMI1640 media on 35 mm Cellstar^®^ cell culture dishes. Prior to irradiation, cells were washed 3x with phosphate-buffered saline (PBS) and replaced with fresh serum-free media. The 6 MV irradiation of LNCaP cells with and without PSMA-targeted AuNPs was performed at Sylvester Comprehensive Cancer Center (Miller School of Medicine, University of Miami) using a Varian Trilogy Clinac (Varian Medical Systems, Inc., Palo Alto, CA, USA). The cell dishes containing ~10^6^ LNCaP cells under 6 mm medium were positioned with source-to-surface distance (SSD) of 1 m (as shown in Figure 1A).

Cell dishes were placed on top of 4 cm solid water for sufficient backscatter and covered by solid water with varying thickness (i.e., 2.5 cm, 10 cm, 20 cm, 30 cm) (Figure 1B,D). The cell dishes themselves were encased in Elasto-Gel bolus to eliminate air gaps and ensure the dishes were not crushed under the weight of the solid water (Figure 1C). Cells were irradiated with a dose rate of 0.6 Gy min^−1^ for doses of 2 Gy, 4 Gy, 6 Gy, and 8 Gy at different depths. Optically stimulated luminescent dosimeters (OSLD) were used to verify the dose delivery accuracy prior to cell irradiation. Following the X-ray irradiation, LNCaP cells were seeded at low density into 100 mm cell culture dishes containing 10 mL of RPMI-1640 medium. Each independent dish was seeded in triplicate sets corresponding to each cell radiation dose and depth. After 3 weeks, each dish with adequately sized colonies was washed with 5 mL PBS and fixed with 10% formalin for 0.5 h, then stained with a solution of 0.5% crystal violet solution (Millipore-Sigma, Burlington, MA, USA). The plating efficiency (PE) was determined by dividing the number of surviving colonies by the original seeding number. The mean value and standard variation of PE was calculated for each treatment group. The survival fraction was calculated as the ratio between the PE of irradiated cells and the PE of non-irradiated cells.

### 2.2. Monte Carlo Simulations

**Figure 2:**
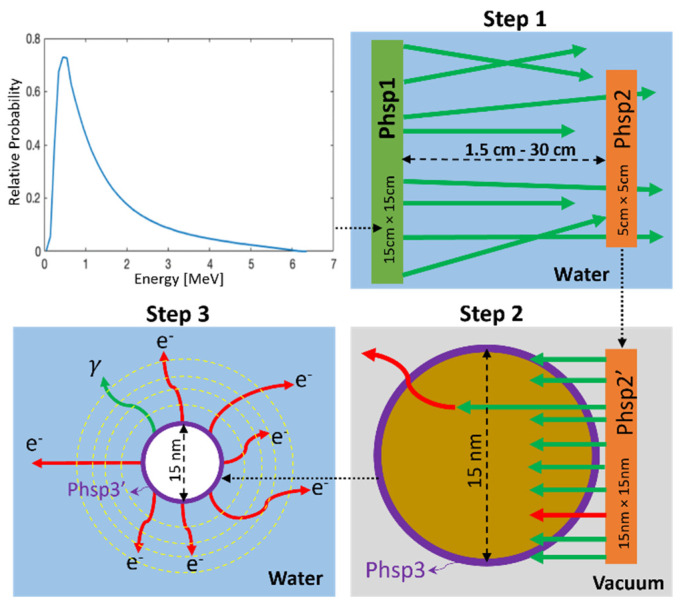
Modular three step multi-scale Monte Carlo (MC) simulated geometry used to determine Gold nanoparticle (AuNP) mediated dose enhancement ratio (DER). Note: red tracks = electrons, and green tracks = photons.

Monte Carlo (MC) simulations were performed using TOPAS version 3.6.p3, which is layered on top of Geant4 version 10.6.p1. The MC simulations were split into three distinct steps based on phase space (phsp) files to increase the simulation efficiency. The schematic setup of the MC simulations is illustrated in Figure 2. The incident photon spectrum from the 6 MV Varian LINAC was downloaded as a phsp file from the International Atomic Energy Agency (IAEA) website, which has been verified via experimental measurements of percent depth dose curve and crossline/inline beam profiles [37].

In the first step, the initial 10 cm × 10 cm 6 MV phsp source file was resized to 15 cm × 15 cm (phsp1) and used as a parallel beam on the surface of a water phantom (40 × 40 × 40 cm^3^). For this step, the CH physics model Livermore was selected for the simulation of MV photons transport in the water phantom, in view of its high performance in low-energy particle tracking [38]. In our simulation, the production energy cut value for electrons and photons was kept as default 250 eV, and the step size is automatically selected by TOPAS using logic from Geant4 that take into consideration the local geometry and physics. Phsp1 contained ~4.7 billion particles, however, it was sampled until the number of original particle histories reached 10 million to reduce the simulation time. Position, momentum, and energy of each particle which crossed a 5 cm × 5 cm scoring plane (phsp2) placed perpendicular to and centered on the beam central axis were recorded. To ensure the accuracy of phsp2 files, the dimensions of phsp1 were set much larger than phsp2, which satisfies charged particle equilibrium (CPE). Moreover, by keeping the phsp1 size much larger than the range of secondary electrons, secondary particle equilibrium (SPE) was satisfied [39]. Several of these phsp2 scoring planes were placed at various distances (2.5 cm–30 cm) from phsp1. About 1.5–4.3 million particles were scored in phsp2 files for the scenarios of different depths.

In the second step, microscopic beam source phsp2′ (15 nm × 15 nm) was obtained by scaling down the macroscopic phsp2 (5 cm × 5 cm) from Step 1. The particle momentum directions in phsp2′ were adjusted to be along with the beam primary axis, by reweighting each particle with 1/cosθ where θ was the angle between the original phsp2′ particle direction and the beam primary axis direction. This maximized the interaction probability of incoming particles with the AuNP while accounting for the contributions of laterally scattered electrons. The spectra in microscopic phsp2′ was used as parallel 15 × 15 nm^2^ beams to irradiate a single AuNP in a vacuum box of 20 × 20 × 20 nm^3^. The original number of particles in phsp2′ is in the order of millions. To ensure a sufficiently high number of particles interacting with the AuNP, phsp2′ was sampled until the number of original particle histories reached 1 billion. The secondary electrons produced through ionization and excitation interactions in the AuNP were scored in a phase space 3 (phsp3) at the outer surface of the nanoparticle. About 110,000–130,000 particles were scored in phsp3 files for the scenarios of different depths. Dose delivered to the AuNP was also recorded during this step, which will be used later when calculating dose enhancement ratio (DER). For this step, CH physics model Livermore, with secondary particle production threshold set at 10 eV, was utilized to track the low-energy secondary particles. Although the recommended applicability limit for electrons in Livermore physics is 250 eV, Livermore physics with 10 eV production cut has been validated with a good agreement with TS physics model for water [40].

In the third step, to ensure statistical significance, the phsp3 recorded previously was sampled until the number of original histories reached 500,000 on phsp3′, which was placed at the center of a 20 × 20 × 20 µm^3^ cube and used as a source to calculate the nanoscopic dose in water. The TS physics model g4em-dna, which tracks electrons down to an energy of 7.4 eV, was utilized to achieve high accuracy and resolution of low-energy electron tracking in water [28,41,42]. The step size for all particles in g4em-dna was set to 1 nm in this step. The dose to water was scored in spherical shells surrounding the AuNP, the first 200 shells were 1 nm thick and the next 200 shells were 50 nm thick for a maximum detection range of ~10 µm.

To obtain the dose enhancement ratio, Steps 2 and 3 were repeated by replacing the AuNP with a water nanoparticle (WNP) of the same size. All the sample numbers for the phase spaces phsp2′ and phsp3′ were kept the same as that for the AuNP scenario, and the physics models employed in each step were also kept the same.

### 2.3. Calculating Dose Enhancement Ratio

The dose enhancement ratio (DER(r)) in terms of radial distance, r, was calculated as the ratio between the deposited dose in water in presence of a single AuNP and WNP. Several normalization steps are essential prior to DER calculation, in view of the number of particles in phase spaces 2 and 3, phsp2 and phsp3, were different for scenarios of AuNP and WNP located at different depths. For example, the nanoscopic source phsp2′ in Step 2 was obtained by sampling phsp2 1 billion times, yet phsp2 files in Step 1 contained different number of particles for different depth scenarios. By dividing the number of particles in phsp2′ by that in phsp2 file, the first normalization factor ($f_{1}$) was calculated at each depth. Similarly, the low-energy secondary electron source phsp3′ in Step 3 was obtained by sampling phsp3 500,000 times, yet phsp3 files in Step 2 contained different number of particles for AuNP and WNP at each depth. By dividing the number of particles in phsp3 file by that in phsp3′, the second normalization factor ($f_{2}$) was calculated at each depth. It is worth noting, the deposited radial dose scored in Step 3 is only induced by the secondary particles produced in the AuNP (or WNP), with no dose coming from the primary beam. This dose from the primary beam, called ‘background dose’ from here on out, is essential to calculating realistic dose enhancement ratios. In this study, the dose deposited in the WNP volume in Step 2 was used as the background dose ($D_{bg}$) at each depth. Based on the aforementioned normalization steps, the ***DER(r)*** was calculated as:

(2) $$DER\left(r\right)=\frac{D_{AuNP}\left(r\right)\times f_{1,depth}\times f_{2,AuNP}+D_{bg}}{D_{WNP}\left(r\right)\times f_{1,depth}\times f_{2,WNP}+D_{bg}}$$

where $D_{AuNP}\left(r\right)$ and $D_{WNP}\left(r\right)$ are the radial dose obtained in Step 3, $f_{1,depth}$ is the normalization parameter $f_{1}$ for irradiation at different depths, $f_{2,AuNP}$, and $f_{2,WNP}$ are the normalization parameters $f_{2}$ corresponding to AuNP and WNP, respectively.

### 2.4. Cell Models

**Figure 3:**
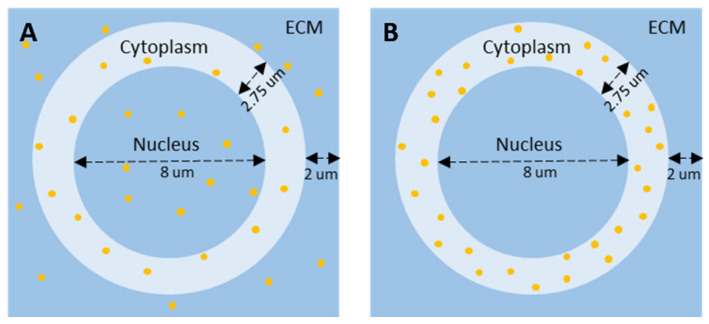
Schematic diagrams of cell with different AuNP biodistribution. (**A**) Homogenous cell model. (**B**) Cytoplasm cell model.

To quantify the effect of PSMA-targeted AuNPs present in the LNCaP cells under 6 MV irradiation, a comparison was made between two cell models, i.e., ‘homogenous model’ in which AuNPs locations were randomly placed throughout the entire cell and extracellular matrix (ECM), and ‘cytoplasm model’ in which AuNP locations were limited to within the cell cytoplasm. As widely used in MC AuNP radiosensitization analysis [43,44], a simplified spherical shaped cell with a centrally located nucleus was employed in this study to evaluate the effect of depth on the cell survival in the presence of AuNPs. The choice of cell and nucleus size was based on the collected TEM image of a single LNCaP cell. Both cell models consisted of a circular 13.5 µm cell diameter with a concentric circular 8 µm diameter nucleus placed in a 17.5 µm × 17.5 µm extracellular matrix (ECM). The homogenous cell model is shown in Figure 3A, and the cytoplasm cell model is shown in Figure 3B. To calculate the microscopic dose enhancement due to the presence of AuNPs within the model, the radial DERs were used as dose point kernels and placed in cell/ECM. The AuNP-induced micro-dosimetry was calculated in two steps under the assumption that the AuNP distribution in three dimension is similar to that of the two-dimension model. First, the entire cell model was given a prescribed homogenous base dose (2, 4, 6 or 8 Gy). Second, the base dose around the position of each AuNP was multiplied by the radial dose enhancement ratio, i.e., the dose enhancement dose point kernels.

### 2.5. Local Effect Model

To model AuNP-assisted radiosensitization, an approach based on the LEM was applied to quantify the effect of highly inhomogeneous dose distributions at the sub-cellular scale induced by AuNPs. The nucleus, located at the center of the cell, was selected as the radiation target for survival fraction calculation herein. The basic hypothesis of the LEM is that the same local dose on a sub-cellular scale results in the same local damage, independent of the energy and type of radiation. LEM relates energy deposition on the nanoscale to the cell survival, expressed as a function of the number of lethal events following the Poisson distribution. The macroscopic survival fraction in presence of AuNPs ($S_{\mathrm{AuNP}}$) can be described as:

(3) $$S_{\mathrm{AuNP}}\left(D\right)=e^{-\bar{N}\;}$$

where $\bar{N}$ is the average number of lethal events in the radiation sensitive target, given by:

(4) $$\bar{N}=-\int_{V_{S}}\;\frac{\ln\left(S_{x}\left(D\right)\right)}{V_{S}}dV$$

where $V_{S}$ is the volume of radiation sensitive target. The average number of lethal events in the sensitive target is calculated as an integral of the number of events locally determined by a linear quadratic model (LQM) dose–response curve $S_{x}\left(D\right)$. Since high-dose response is overestimated in the LQM model, a threshold-based model was used to determine the dose response curve:

(5) $$S_{x}\left(D\right)=\{\begin{array}{ll} e^{-\alpha D-\beta D^{2}} & :\left(D\leq D_{t}\right)\\ e^{-\alpha D-\beta D^{2}+S_{max}\left(D-D_{t}\right)} & :(D>D_{t}) \end{array}$$

where $D_{t}$ is a tunable parameter (threshold dose) and $S_{max}=\alpha +2\beta D_{t}$. In this study, the parameters *α* and *β*, which relate local dose to $S_{x}$, were determined experimentally by curve fitting the in vitro cell survival fraction $\left(S_{x}\right)$ of LNCaP cells treated with radiation (without AuNPs). The intracellular dose distribution, *D*, in presence of AuNPs, was determined by combining the nanoscopic dose enhancement ratio of AuNPs as calculated in the MC simulations (in Section 2.3) and the cell geometry models with specific AuNP biodistribution (in Section 2.4). The calculated dose distribution was then used on the LEM (Equations (3)–(5)) to calculate the cell survival fraction.

### 2.6. Sensitization Enhancement Ratio

To quantitatively compare the survival fraction curves obtained from in vitro experiments with that simulated based on MC-LEM, the sensitization (sensitizer) enhancement ratio (SER) was employed in this study. ICRU Report 30 suggests when comparing survival fraction curves that the entire curve is taken into account, as opposed to a single point along the curve [45]. SER is expressed in terms of mean inactivation dose (MID), which is the area under the curve (AUC) of the survival fraction curve [46]:

(6) $$MID=\int_{0}^{\infty }S_{x}\left(D\right)dD$$

With the calculated MIDs for the radiation survival curves with and without AuNPs, the AuNP-induced SER was calculated as [46]:

(7) $$SER=\frac{MID_{\mathrm{IR}}\;}{MID_{\mathrm{IR}+\mathrm{AuNP}}}$$

## 3. Results

### 3.1. Characterization of PSMA Targeted AuNPs

**Figure 4:**
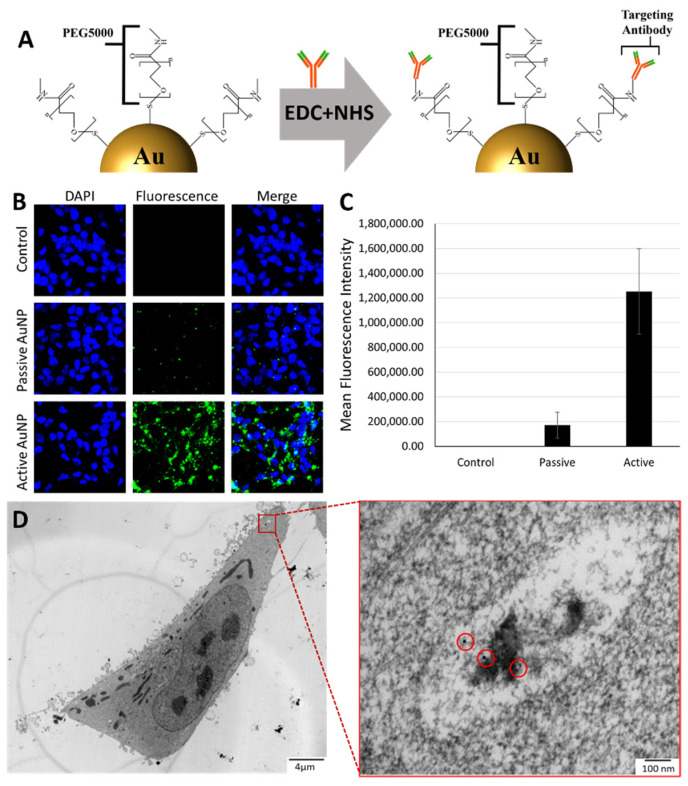
(**A**) Schematic structure of PSMA targeted AuNP. (**B**) Fluorescence imaging showing AuNP targeting and uptake. (**C**) Quantitative analysis of fluorescence microscopy images via fluorescence counts. (**D**) Transmission electron microscope (TEM) images of LNCaP cancer cells treated with AuNPs.

Figure 4A shows the schematic structure of the PSMA targeted AuNP. The anti-PSMA antibodies were conjugated to AuNP via EDC-NHS chemistry to enable active targeting of LNCaP prostate cancer cells. For the passive-targeting control, the same amount of IgG antibodies, replacing anti-PSMA antibodies, were conjugated to AuNP with the same EDC-NHS chemistry. The uptake and targeting efficiency of PSMA targeted AuNPs on LNCaP cells was verified through fluorescence microscopy imaging (Figure 4B). Quantitative analysis of fluorescence microscopy images, based on the fluorescence counts, showed the higher binding of active-targeting AuNPs in LNCaP cells compared with the passive-targeting AuNPs (as shown in Figure 4C). The TEM images of LNCaP cells treated with PSMA targeted AuNPs further validated the internalization of AuNPs with the help of active targeting (Figure 4D). By weight, there were about 10 picograms of AuNPs per LNCaP cell.

### 3.2. In Vitro Clonogenic Assay

**Figure 5:**
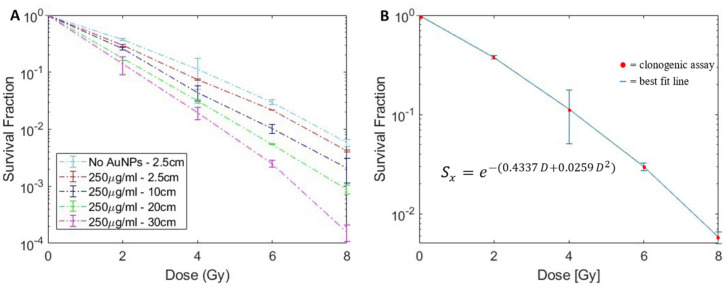
(**A**) Survival fraction curve of LNCaP cells treated with 250 µg/mL PSMA-AuNPs irradiated in vitro at various depths in water. (**B**) Survival fraction curve fitting of LNCaP cells without AuNPs for LQM *α* and *β* values.

As demonstrated in our previous study, AuNPs with concentration of 250 µg/mL did not produce any intrinsic toxicity towards LNCaP cells [12,47]. Figure 5A shows the clonogenic surviving fraction of LNCaP cells following irradiation with clinical 6 MV radiation beams with PSMA targeted AuNPs (250 µg/mL) at depths of 2.5, 10, 20 and 30 cm. As the control group, the 6 MV irradiation without AuNPs was performed at a depth of 2.5 cm (Figure 5B). AuNP-induced radiosensitization of LNCaP cells enhanced with increasing depth, as expected. As a result, cell survival fraction was reduced across all doses as depth was increased. The *α* and *β* parameters, obtained by fitting the no-AuNP-2.5 cm surviving fraction curve with the LQM model, are shown in Figure 5B. The goodness of fit was evaluated through an R-squared value of 0.999. The *α* and *β* parameters, corresponding to irradiation with 250 µg/mL AuNP at different depths, are shown in Table 1. As shown, the primary effect of increasing depth of AuNP treated LNCaP cells was on the *α* parameter, which dominates at low doses and contributes to the more linear surviving fraction curve and is related to unrepairable double strand DNA breaks [48].

### 3.3. Characterization of Depth Effects on Energy Spectrum

**Figure 6:**
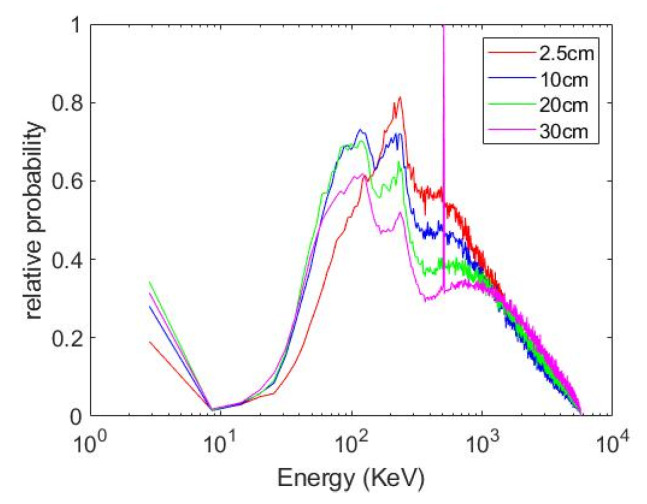
Megavoltage (MV) phase space files collected at various depths in water.

The MC generated energy spectrum of the irradiation beam propagating through water of different depths are shown in Figure 6, plotted as relative probability vs. energy. The detailed characteristics of the MV beams collected at different depths are summarized in Table 2. The mean and median energy increase with depth due to the beam hardening. For instance, as the depth increased from 2.5 cm to 30 cm, the mean and median energy experienced 21% and 35% increase, respectively. However, MV photon beams contain an increasing percentage of keV particles (including photons and electrons) when penetrating water of different depths. The increased percentage of low-energy particles contributes to the local dose enhancement around AuNPs.

**Figure 7:**
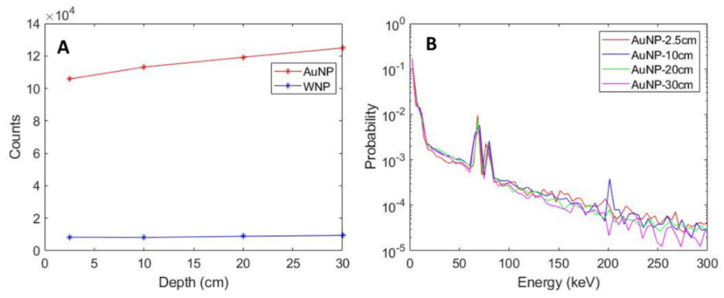
Secondary particle count and energy spectrum at various depths collected on phsp3. (**A**) shows the secondary particle counts for both the AuNP and the water nanoparticle (WNP). (**B**) shows the energy spectrum of secondary particles of AuNP.

The number of secondary particles generated from AuNPs under different depths, depicted as phase space 3 (phsp3) in Step 2 of the MC simulation (as shown in Figure 2), is shown in Figure 7A. The spectra of AuNP-induced secondary particles plotted as an energy histogram is shown in Figure 7B. The characteristics of these secondary particle spectra are detailed in Table 3. As the depth of the AuNPs increased, the number of secondary particles scored increased relative to incident particle count, which is attributed to the increased low-energy component in the incident spectrum due to the scattering accumulated along the penetration path. However, the secondary spectra count for the WNP remained relatively constant, as shown in Figure 7A. The mean and median energy, and the proportion of particles under 100 keV remained relatively constant as well, as seen in Figure 7B and in Table 3. Au K-edge emission can be seen on Figure 7B at ~68.8 keV, ~66.9 keV, and ~77.9 keV for $K_{\alpha 1}$, $K_{\alpha 2}$, and $K_{\beta 1}$, respectively.

### 3.4. AuNP Micro-Dosimetry

**Figure 8:**
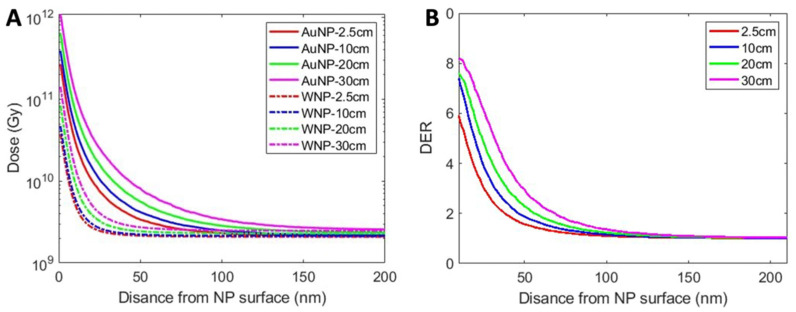
(**A**) Dose as a function of radial distance for both AuNPs and WNPs. (**B**) Dose enhancement ratio (DER) as a function of radial distance.

The radial dose distribution and DER produced around a single AuNP located at different depths is shown in Figure 8. Figure 8A also compares the radial dose distribution originating from AuNP and WNP. Due to the enhanced number of low-energy secondary electrons, AuNP generated higher dose than WNP at the same radial distance. As the distance from the AuNP surface increased, the dose originating from the AuNP decreased rapidly. The dose induced by AuNPs located at greater depths was higher than that by AuNPs at shallower depth. This is due to the higher number of secondary particles generated by AuNPs modeled at greater depths. Figure 8B reveals that the DER is higher at deeper depth of irradiation, which can be attributed to the higher percentage of low-energy particles.

### 3.5. Monte Carlo LEM (MC-LEM) Model

**Figure 9:**
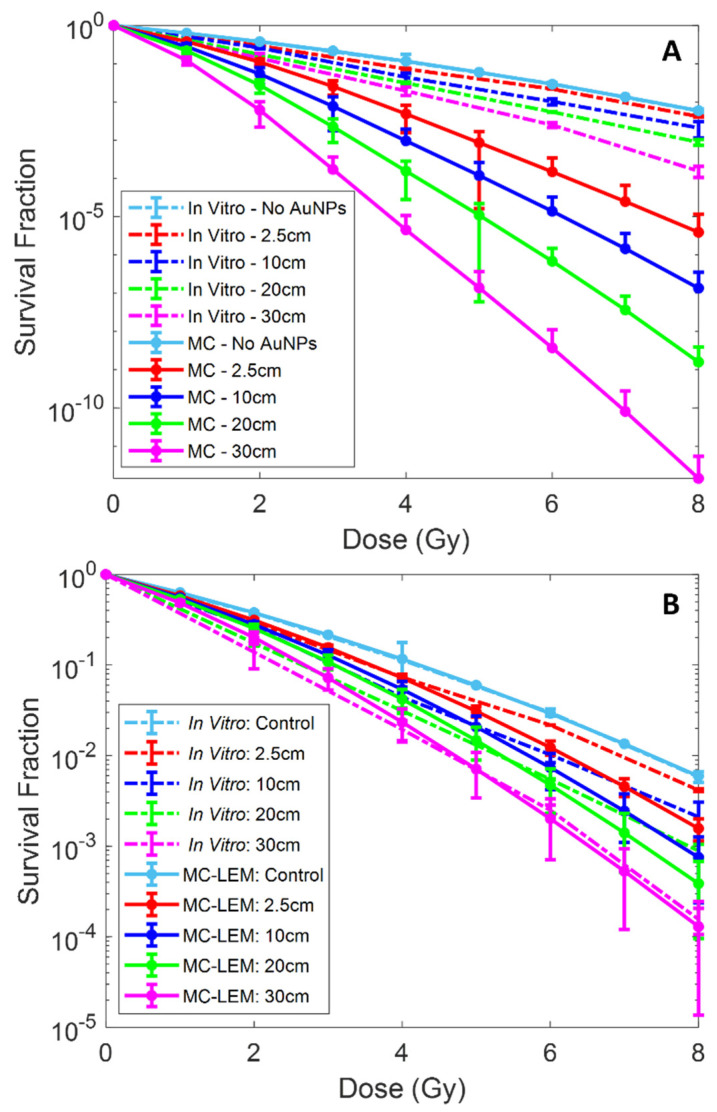
Monte Carlo (MC) and local effect model (LEM) calculated survival fraction curves at various depths for 10 pg/cell AuNPs using ‘homogenous’ model, (**A**), and ‘cytoplasm’ model, (**B**). Additionally, the in vitro clonogenic assay derived survival fraction curves are shown at corresponding depths for comparison.

The expected surviving fraction of LNCaP cells with PSMA targeted AuNPs using the LEM was calculated from the MC-simulated radial DER profiles. The resulting MC-LEM survival fraction curves of cells located at different depths are shown in Figure 9, compared to the in vitro experimental data. Figure 9A,B present the MC-LEM survival fraction curves based on the homogenous cell model (Figure 3A) and cytoplasm cell model (Figure 3B), respectively. As expected, the MC-LEM calculated surviving fractions decreased with increasing depth of irradiated cells. Compared to the homogenous cell model, the cytoplasm cell model resulted in survival fraction curves with better fit to the in vitro survival fraction curves. We attribute this result to the AuNP distribution for the cytoplasm model matching more closely with the observed distribution on TEM (Figure 4D). Table 4 shows a comparison of the SER calculated from survival fraction curves corresponding to in vitro experiments, MC-LEM homogenous and cytoplasm models. The SER results reaffirm that the cytoplasm model resulted in better agreement with in vitro results, with an average difference in mean SER < 5%.

**Figure 10:**
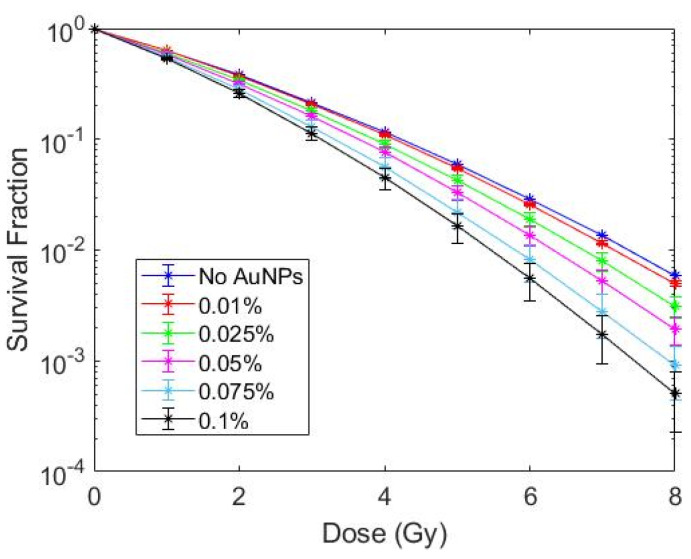
Survival fraction curves determined using MC-LEM method at 2.5 cm depth for varying concentrations of AuNPs in the cytoplasm model.

Upon confirming that the cytoplasm cell model produced accurate results, this model was tested at various concentrations of AuNPs ranging from 0.1% to 0.01% AuNP by weight at a depth of 2.5 cm. Survival fraction curves at each concentration are shown in Figure 10. As expected, SER increased with increasing Au concentration as shown in Table 5, with minimal sensitization enhancement at 0.01% Au by weight. 

## 4. Discussion

Active targeting techniques afford increased accumulation of AuNPs within tumors; furthermore, actively targeted AuNPs are internalized selectively within cancer cells, resulting in a higher therapeutic ratio. AuNP mediated radiosensitization has demonstrated great potential under kilovoltage irradiation in both external superficial therapy (e.g., 100–500 kVp) and internal brachytherapy (e.g., ^125^I, ^169^Yb) [49,50]. However, AuNP enhanced radiation therapy under megavoltage energies have been shrouded in uncertainty and contradiction due to the disparities between the calculated macroscopic dosimetry and the radiosensitization effects observed in in vitro and in vivo experiments [24,49,51]. The aim of this study was to investigate the feasibility of AuNP-aided prostate cancer radiation therapy by quantifying radiosensitization of PSMA-targeted AuNPs under megavoltage radiation energy at clinically relevant depths. AuNP mediated radiosensitization irradiated with a 6 MV photon beam at various depths was first demonstrated through clonogenic assays in vitro. Monte Carlo AuNP micro-dosimetry combined with the LEM on two cell models with different AuNP biodistribution patterns were then utilized to quantify the effects of AuNP location and concentration on cell surviving fraction. The in vitro results validated that AuNP-induced radiosensitization of LNCaP cells under 6 MV energy increased with increasing depth. Furthermore, our results show that MC-LEM model produced a good correlation to experimental AuNP radiosensitization, when MC AuNP biodistribution mirrored the experimentally observed biodistribution. We have demonstrated the highly active targeting of the developed PSMA-targeted AuNP on the LNCaP cells, which were established from a lymph node metastasis of a Caucasian patient with metastatic prostate cancer. Further studies are required to investigate the targeting efficiency and radiosensitization, of the developed PSMA-targeted AuNPs, for prostate cancer at different stages.

The reliability of calculated micro-dosimetry and DER as a function of radial distance from the AuNP surface was critical to quantifying AuNP radiosensitization. In the present study, the maximum DERs of 5.5, 7, 7.5 and 8 were obtained at depths of 2.5 cm, 10 cm, 20 cm and 30 cm, respectively. Tsiamas et al. calculated DER of 10 and 100 nm diameter AuNPs under 6 MV radiation at a depth of 0 cm, 2 cm, 10 cm, and 20 cm using GEANT4 based MC simulations and found DER increased with increasing depth with a maximum DER of ~4.2 at 20 cm depth [52]. Lin et al. evaluated AuNP-mediated radiosensitization for a 6 MV photon source using MC simulations on TOPAS and calculated an increasing DER with increasing radial distance [8]. However, the DER vs. radial distance plots presented in Lin et al. neglected normalization and background factors, highlighting the importance of these factors in creating realistic dose enhancement ratios. As shown, the radial dose and DER distributions calculated herein is close to those in previously peer-reviewed publications, with small differences attributed to MC code differences with respect to electron transport simulation, associated electron interaction cross sections, AuNP size, and other geometry model considerations.

When translating MC micro-dosimetry to in vitro experimental results, the location of AuNPs within the cell were shown to drastically change the predicted sensitization in terms of SER. The LEM relies on a user specified sensitive region(s) to score damage. In this study, the cell nucleus with diameter of 8 µm was used as the sensitive region. The homogenous cell model placed AuNPs randomly throughout the entire cell (including nucleus) and surrounding ECM. This model resulted in an overestimation of cell killing due to the AuNPs located within nucleus, indicating that improved radiotherapy response can be achieved by nuclear targeting of AuNPs. The cytoplasm model, which limited AuNPs to the cell cytoplasm, matched well with the collected TEM image, where no AuNP was observed in the nucleus. The distribution of AuNPs must be considered carefully as it is known to differ between cell lines and nanoparticle functionalization. For example, in 9 LGS cell line, AuNPs clustered in the cytoplasm, which causes superimposing and shielding effects on the AuNP dose enhancement [53].

A major component to this study was evaluating the effect of depth on beam quality/energy. It was shown in Figure 6 and Table 2 that as the 6 MV beam traversed water, the proportion of low energy particles within the beam increased. This increase in low energy photons at AuNP depth directly contributed to an increase in secondary particle production and an increase in DER. This effect is amplified when using a flattening filter free (FFF) beam. A MC simulation study by Tsiamas et al. found that FFF 6 MV beams produced nearly double the DER compared to standard flattened beams [52].

As previously mentioned, the local effect model accounts for damage to a user specified sensitive area (or volume), which was chosen to be the cell nucleus. While the cell nucleus is undoubtedly a sensitive target, the mitochondria and other cell organelles have been shown to play a significant role in determining cell survival following radiation [54,55]. Notably, mitochondria are the only non-nuclear location where DNA resides and can account for 30% of total cell volume [56]. Ignoring damage to mitochondria (and other organelles) may result in under estimations of cell death following radiation.

Cell coatings and surface functionalization such as polyethylene glycol (PEG) and PSMA targeting agents, respectively, are vital to limiting AuNP clustering (and biocompatibility) and increasing cellular uptake, respectively. However, these modifications to the AuNP may also influence the secondary particle production/escape. Although PEG coatings are typically very thin, on the order of nanometers, and can capture/attenuate some of the secondary particles produced within the AuNP. A MC simulation study by Peukert et al., which tested the effects of AuNP size and coatings on dose using a proton source, found that PEG coating density had a minimal effect on secondary particle production [57]. Thus, the effects of self-shielding due to PEG and PSMA was not considered in this study.

As demonstrated by our results, the LEM prediction of cell survival is highly dependent on the specific AuNP distribution. In the present study, a spherical shaped cell with a centrally located nucleus was employed, which is a simplified model. Sung et al. concluded that geometric parameters such as the shape, size, and location of the cell and the nucleus are critical for the AuNP radiosensitization [31]. Therefore, the effects of these geometric parameters on the AuNP-assisted MV radiosensitization will be performed in the future. Moreover, modelling AuNP enhanced radiotherapy by means of MC-LEM with accurate physics models is crucial to assist the development of tumor targeted radiosensitization paradigms. Sophisticated MC-LEM models should also incorporate the biological and chemical effects caused by AuNPs, including increased reactive oxygen species (ROS) production, oxidative stress, repair inhibition and more [4,18]. A study by Rudek et al., showed that ROS significantly increases in the presence of AuNPs [32]. This ROS increase is expected to increase DNA damage and thus reduce survival fraction. MC results presented here without AuNP effects on ROS production (and other chemical and biological effects) are conservative estimations on the overall AuNP mediated radiosensitization.

## 5. Conclusions

Development of an accurate computational model for AuNP enhanced radiotherapy is essential to enable treatment planning and hence the progression of AuNP-mediated RT into clinical use. In this study, the clinical feasibility of AuNP radiosensitization on prostate cancer was investigated through in vitro and in silico experiments by quantifying radiosensitization of PSMA-targeted AuNPs under 6 MV irradiation at various clinically relevant depths. The in vitro clonogenic assay results validated the AuNP-mediated radiosensitization of prostate cancer using the clinical LINAC source. The good agreement between the in silico simulated and in vitro measured surviving fractions demonstrated the accuracy of the Monte Carlo based local effect model (MC-LEM). Based on the real AuNP biodistribution obtained with transmission electron microscopy (TEM), MC-LEM will be an essential platform for quantifying AuNP-induced radiosensitization, benefiting the design of multifunctional AuNPs for radiotherapy.

## Figures and Tables

**Table 1:** LQM *α* and *β* values extracted by curve fitting the clonogenic assay derived survival fractions at each depth for AuNP treated and untreated LNCaP cells in vitro. Values are presented with 95% confidence intervals.

Concentration and Depth	α (Gy^−1^)	β (Gy^−2^)
No AuNPs—2.5 cm	0.4337 ± 0.068	0.0259 ± 0.008
250 µg/mL—2.5 cm	0.5800 ± 0.244	0.0123 ± 0.029
250 µg/mL—10 cm	0.7550 ± 0.283	0.0028 ± 0.034
250 µg/mL—20 cm	0.8518 ± 0.051	0.0030 ± 0.006
250 µg/mL—30 cm	0.8370 ± 0.406	0.0300± 0.049

**Table 2:** Characteristics of megavoltage (MV) beam collected at different depths as plotted in Figure 6. E_mean_ and E_median_ are the mean and median energy, respectively. E < 100 keV is the percent of particles in the beam with energy less than 100 keV. Additionally, shown is the percentage of electrons contained in the beam and the total number of particles (Count) at each depth.

Depth (cm)	E_mean_ (MeV)	E_median_ (MeV)	E < 100 keV (%)	Electrons (%)	Count (Million)
2.5	1.53	1.16	2.26	1.17	1.485
10	1.53	1.16	3.60	1.19	1.151
20	1.67	1.34	3.79	1.33	0.715
30	1.85	1.57	3.82	1.46	0.431

**Table 3:** Spectrum characteristics of secondary particles emitted from AuNPs located at different depths, with the same number of incident photons.

Depth (cm)	Number of Particles Scored	E_mean_ (KeV)	E_median_ (KeV)	E < 100 keV (%)
2.5	110,674	22.04	1.14	96.76
10	119,188	22.13	1.30	96.98
20	124,832	21.63	1.24	97.23
30	130,168	21.40	1.12	97.47

**Table 4:** Sensitization enhancement ratio (SER) calculated on MC-LEM survival fraction curves for both homogenous and cytoplasm cell models, compared against that from in vitro clonogenic assay.

		SER	
Depth (cm)	Homogenous Model	Cytoplasm Model	In Vitro
2.5	1.92 ± 0.12	1.16 ± 0.03	1.14 ± 0.03
10	2.34 ± 0.23	1.26 ± 0.06	1.25 ± 0.03
20	2.62 ± 0.17	1.34 ± 0.09	1.43 ± 0.04
30	3.12 ± 0.17	1.52 ± 0.12	1.55 ± 0.05

**Table 5:** Sensitization enhancement ratio (SER) calculated from Figure 10.

Gold Weight (%)	SER: MC-LEM
0.01%	1.02 ± 0.01
0.025%	1.09 ± 0.03
0.05%	1.15 ± 0.04
0.075%	1.26 ± 0.07
0.01%	1.32 ± 0.07

## Data Availability

Not applicable.
